# Water Stress and Aphid Feeding Differentially Influence Metabolite Composition in *Arabidopsis thaliana* (L.)

**DOI:** 10.1371/journal.pone.0048661

**Published:** 2012-11-07

**Authors:** Inga Mewis, Mohammed A. M. Khan, Erich Glawischnig, Monika Schreiner, Christian Ulrichs

**Affiliations:** 1 Department of Quality, Leibniz-Institute of Vegetable and Ornamental Crops Großbeeren/Erfurt e.V., Großbeeren, Germany; 2 Division Urban Plant Ecophysiology, Institute of Horticultural Sciences, Humboldt-Universität zu Berlin, Berlin, Germany; 3 Lehrstuhl für Genetik, Technische Universität München, Freising, Germany; RIKEN Plant Science Center, Japan

## Abstract

Little is known about how drought stress influences plant secondary metabolite accumulation and how this affects plant defense against different aphids. We therefore cultivated *Arabidopsis thaliana* (L.) plants under well-watered, drought, and water-logged conditions. Two aphid species were selected for this study: the generalist *Myzus persicae* (Sulzer) and the crucifer specialist *Brevicoryne brassicae* (L.). Metabolite concentrations in the phloem sap, which influence aphid growth, changed particularly under drought stress. Levels of sucrose and several amino acids, such as glutamic acid, proline, isoleucine, and lysine increased, while concentrations of 4-methoxyindol-3-ylmethyl glucosinolate decreased. *M. persicae* population growth was highest on plants under drought stress conditions. However, *B. brassicae* did not profit from improved phloem sap quality under drought stress and performed equally in all water treatments. Water stress and aphids generally had an opposite effect on the accumulation of secondary metabolites in the plant rosettes. Drought stress and water-logging led to increased aliphatic glucosinolate and flavonoid levels. Conversely, aphid feeding, especially of *M. persicae*, reduced levels of flavonoids and glucosinolates in the plants. Correspondingly, transcript levels of aliphatic biosynthetic genes decreased after feeding of both aphid species. Contrary to *M. persicae*, drought stress did not promote population growth of *B. brassicae* on these plants. The specialist aphid induced expression of *CYP79B2*, *CYP79B3*, and *PAD3* with corresponding accumulation of indolyl glucosinolates and camalexin. This was distinct from *M. persicae*, which did not elicit similarly strong camalexin accumulation, which led to the hypothesis of a specific defense adaptations against the specialist aphid.

## Introduction

In their natural growing environment, plants encounter multiple environmental stresses that can alter both their chemical composition and the associated herbivore community. Drought is a major environmental stress that not only directly influences plant physiological processes leading to decreased growth, but also changes the allocation of resources and profile of secondary metabolites [1], [2], [3]. Enhanced levels of soluble proteins [1], [4], soluble carbohydrates [5], [6], and free amino acid [7], [8] in plants caused by drought may favor the population growth of phloem-feeding insects. Increased population size on drought-stressed plants is observed for some aphid species [2], [3], [9], [10]. Population growth of the generalist *Myzus persicae* (Sulzer) was more rapid on drought-stressed plants; however, the crucifer specialist *Brevicoryne brassicae* (L.) performed equally well on drought and well-watered *Brassica oleracea* var. *italica* (broccoli) plants [2], [3].

Besides the general nutritional quality of phloem sap (primary metabolites), defensive compounds of host plants such as glucosinolate may be responsible for such a variation in the aphids’ performance on drought-stressed plants. Decreased levels of glucosinolates in drought-stressed brassicaceous crops in connection with the improved performance of generalist aphids has been reported in previous studies [2], [3], [11]. Unlike generalist aphids such as *M. persicae* [12], crucifer specialists such as *B. brassicae* may tolerate to some extent glucosinolates in their host plants. However, the influence of drought stress on secondary metabolite accumulation in relation to the impact this has on plant resistance against aphids with different feeding specialization is still not well understood.

The glucosinolate-myrosinase system is the characteristic chemical defense mechanism against herbivores in plants of the order Brassicales. Tissue damage, such as during herbivory, brings glucosinolates in contact with their hydrolyzing enzymes (myrosinases), which are localized in different cells or compartments [13], [14]. Different biologically active compounds such as isothiocyanates and nitriles are released, which are deterrents to many herbivore insects [15]. Aphids are able to avoid the glucosinolate-myrosinase defense in plants since they only inflict minor wounding on the plant while feeding. Intact glucosinolate, but no corresponding hydrolyses product, has been detected in the honey-dew of aphids [16]. Therefore, glucosinolates in phloem sap, and not their hydrolysis products, may provide a defense against aphids. Although aphids inflict slight wounds during feeding, elicitors released by aphids can activate plants’ responses and modify the glucosinolate profile of host plants [3], [17], [18]. A recent study with broccoli plants has shown that the availability of soil water changes the plant’s glucosinolate response to aphid feeding. Glucosinolate levels in water-stressed plants decreased after *M. persicae* feeding, but remained unchanged after feeding by *B. brassicae* [3].

**Figure 1:**
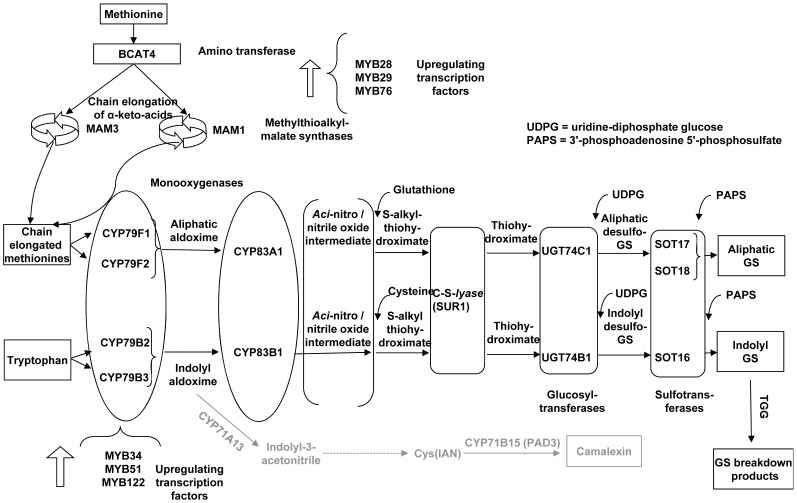
Outline of glucosinolate and camalexin biosynthetic pathways in *A. thaliana* plants. (Grey indicates the last steps of the camalexin pathway independent from glucosinolate biosynthesis; large arrows indicate chain elongation of keto-amino acids, each cycle adds one methyl group).

In *A. thaliana*, the glucosinolate biosynthetic pathway has been well studied and most of the associated genes encoding the required enzymes have been isolated and characterized (Figure 1) [15], [19]. Glucosinolates in plants are synthesized from amino acids and their type depends on the precursor amino acid. The aliphatic glucosinolates are mainly synthesized from methionine, and indolyl glucosinolates from tryptophan. In aliphatic glucosinolate biosynthesis, methionine undergoes chain elongation before entering the core structure pathway. Methionine is initially deaminated by BCAT4 to 2-oxo acid, which enters a cycle of three successive transformations to add a single methylene group, and condensation with acetyl-CoA is catalyzed by MAM1 and MAM3. Chain-elongated methionines are then converted to the corresponding aldoximes by CYP79F1 and CYP79F2, while for the indolyl route CYP79B2 and CYP79B3 accept tryptophan as the substrate. The aldoximes are further metabolized by cytochrome P450 belonging to the CYP83 family (CYP83A1 with a preference for aliphatic and CYP83B1 for indolyl aldoximes) to form S-alkylthiohydroximates with glutathione as the sulfur donor [20]. Thiohydroximates are formed by C-S lyase (SUR1) cleaving S-alkylthiohydroximates. This is followed by glucosylation by glucosyltransferases of the UGT74 family to form desulfo glucosinolates, whereby UGT74C1 glucosylates methionine-derived thiohydroximates; UGT74B1 metabolizes tryptophan-derived thiohydroximates. The final sulfation step is catalyzed by sulfotransferases. Among these, SOT17 and SOT18 metabolize methionine-derived and SOT16 tryptophan-derived precursors. After forming the glucosinolate core structure, secondary modification of the side chain takes place which creates structural biodiversity and changes biological activity [19]. In recent years, transcription factors belonging to the *MYB* gene family have been identified to regulate glucosinolate biosynthesis. *MYB28*, *MYB29*, and *MYB76* control aliphatic glucosinolate biosynthesis [19], [21], [22], [23], [24]; *MYB34* [25], *MYB51*, and *MYB122* [21] regulate indolyl glucosinolate biosynthesis.

Cross-talk between the glucosinolate pathway and other metabolic pathways may have an impact on the actual glucosinolate profile produced [26], e.g. since indolyl acetaldoxime serves as a branching point for indolyl glucosinolate and camalexin biosynthesis (Fig. 1) [27], [28]. The phytoalexin camalexin plays a crucial role in the defense against fungal and bacterial pathogens [29] and accumulates at infection sites [30], [31], [32], [33]. A recent study demonstrated that *A. thaliana* plants accumulate camalexin after infestation with *B*. *brassicae* [34]. However, no comparative study has been undertaken to analyze the camalexin response elicited by the feeding of generalist and specialist aphids. Since drought stress alters indolyl glucosinolate metabolism in plants [2], [3], it is possible that biosynthesis of camalexin in *A. thaliana* plants is also affected by water stress.

In the present study, the model plant *Arabidopsis thaliana* (L.) ecotype Columbia (Col-0) was used to study the genetic regulation of glucosinolate biosynthesis as a response to water stress and aphid feeding. Three water regimes - well-watered, drought, and water-logged - and two important aphid pests - the specialist *B. brassicae* and the generalist *M. persicae* [35] - were used to elucidate the molecular and biochemical response of plants cultivated under different water status conditions to insects. Besides major glucosinolate biosynthetic genes and transcription factors, we analyzed genes related to major plant signaling pathways, and *PAD3* involved in camalexin biosynthesis [36], [37]. The main research objective was to investigate factors that can vary the performance of aphid species with different specializations. Unlike Khan et al. (2), who focused on water stress, and its effects on both aphid performance and accumulation of glucosinolates and flavonoids in broccoli, we undertook a wider approach by using *A. thaliana* and included the molecular level for defensive metabolite biosynthesis. Water stress mediated changes in the nutritional components of phloem sap, such as levels of sugars and free amino acids were analyzed along with flavonoids as secondary metabolites and defensive compounds such as glucosinolate and camalexin.

## Materials and Methods

### Plant Material and Cultivation

*Arabidopsis thaliana* (L.) Columbia wild type (Col-0) was used to study the effect of water stress-induced changes in plant metabolites, corresponding gene expression, and aphid performance. Seeds were sown in pots (10 cm×10 cm) filled with potting substrate (Einheitserde Werkverband e.V., Germany) and stratified for three days at 4°C in the dark. Seven days after germination, the seedlings were transferred to single pots (7 cm×6 cm). Plants were cultivated at 22±1°C, 60±5% relative humidity and a photoperiod of 10 hours with 200 µmol m^−2^ s^−1^ light intensity. The photoperiod was changed from 10 hours to 12 hours with the same light intensity when the experiment with the aphids began. Plants were watered three times per week, with quantities as required, before applying the different water conditions.

### Rearing the Aphids

*B. brassicae* and *M. persicae* were reared on *Brassica rapa* ssp. *chinensis* (pak-choi) plants in the insectarium at the Urban Plant Ecophysiology Division of Humboldt University Berlin, Germany, at 22±1°C temperature and a 14-hour photoperiod. To maintain the aphid population, new plants were added roughly every two weeks, and old plants were removed after the aphids had settled on the new plants.

### Water Stress Treatment and Experimental Procedures

25-day-old *A. thaliana* Col-0 plants were assigned to three different irrigation regimes (treatment): 160 ml water/week (water-logged), 80 ml/week (well-watered) and 40 ml/week (drought stress), each delivered three times per week. Plants were placed on 4 cm deep plastic trays (30 cm wide×60 cm long) to retain any excess water and twenty plants were arranged per tray. After one week of different water treatments, when the plants were 32 days old, ten plants per treatment were harvested in pairs by cutting at the base, then flash frozen in liquid nitrogen and stored at −80°C for chemical analysis. The bioassay with aphids was started in parallel and 15 adult *B. brassicae* or *M. persicae* were released on each *A. thaliana* plant. Each plant was caged individually in a transparent plastic cylinder (6 cm diameter×10 cm height) covered with mesh gauze. The experiment with aphid species was performed with 13 replicates. Control plants of each water treatment were also caged. After one week, ten control plants and 10 aphid-treated plants were harvested in pairs for biochemical analysis, resulting in five replicates per treatment. The remaining three plants per treatment were harvested separately for molecular biological studies. Control plants were cut directly at the base and immediately flash-frozen in liquid nitrogen, whereas aphids were brushed off and counted in the insect treatments. Plant material was stored at −80°C before freeze drying samples for biochemical analysis (ALPHA 2- 4 LD plus, Martin Christ Gefriertrocknungsanlagen GmbH, Germany). Samples were ground using a mixer mill MM 301 (Retsch GmbH & Co. KG, Germany).

An additional experiment, in parallel, with similar water treatments and controlled conditions, as described previously, was conducted to collect phloem sap after one and two weeks of water stress. Collected phloem sap samples were analyzed for sugars, amino acids, and glucosinolates. Furthermore, ten plants per water treatment were cultivated to determine the water content of the soil and the plants using the gravimetric method. The fresh weights of soil and plants were recorded, along with the dry weights after 48 h drying at 105°C in an oven. The water content of soil, expressed by weight as the ratio of the water mass present to the dry weight of the soil sample, determined for well-watered, drought, and water-logged treatments was 77.21%, 59.12%, and 81.93%, respectively. Also the dry weight of plants was recorded after two weeks to estimate plant growth under different water conditions.

### Collection of Phloem Sap

Phloem exudates were collected from *A. thaliana* leaves using the EDTA method according to King and Zeevaart [38]. Upper, completely expanded leaves (4^th^ to 5^th^) were taken from plants cultivated under different water conditions. Phloem exudates were collected twice, after one or two weeks of water stress. Briefly, each leaf was cut at the base, weighed and incubated in 2 ml vials containing 200 µl 15 mM EDTA-disodium salt solution (pH 7.5) for four hours in a humid climate chamber at 20°C. The phloem exudates were then stored at −80°C.

### Analysis of Phloem Amino Acid

Phloem exudates from ten leaves were combined for each replicate and mixed gently. Four replicates per treatment were analyzed for amino acid concentrations. The phloem samples were run on an ARACUS amino acid analyzer (membraPure GmbH, Germany) and the concentration of amino acid was calculated using amino acid standards according to the manufacturers’ protocol (Sigma Aldrich, USA). Tryptophan was detected in the exudates, but levels were too low for reproducible quantitative analysis.

### Analysis of Phloem Sugar

For determination of phloem sugar, ten phloem sap samples were pooled for each replicate, lyophilized and filled up to 200 µl. Four replicates were processed per treatment. Sucrose, D-glucose, and D-fructose were determined in the phloem sap samples using a UV spectrometric method with an Enzymatic BioAnalysis kit (Boehringer Mannheim/R-Biopharm) and following the manufacturer’s instructions.

### Glucosinolate Analysis

Glucosinolates were extracted as described in detail by Mewis et al. [39]. Briefly, a freeze-dried sample (20 mg) was extracted with 70% (v/v) methanol at 80°C in a heat block for 5 min and centrifuged for 5 min at 4000 g. The pellet was re-extracted twice and supernatants were combined. To quantify the glucosinolate content, 60 µl of 1 mM *p*-hydroxybenzyl GS (isolated from *Sinapis alba* seeds) was initially added as an internal standard. To convert the methanol-extracted glucosinolate to the desulfo-form, the extract was loaded onto 9 cm Poly-Prep® Chromatography columns (Biorad, CA, USA) filled with 500 µl of a 10% (w/v) suspension of DEAE Sephadex A-25 in 2 M acetic acid and preconditioned with 6 M imidazole-formate solution in 30% formic acid. The column was washed with 0.02 M sodium acetate buffer (pH 4.0) before adding 75 µl of aryl sulfatase solution (Sigma-Aldrich; H-1 from *Helix pomatia*). The column was incubated overnight. Desulfated glucosinolates were eluted with 1 ml of ultrapure water (Milli-Q quality). Extracts were run on a Dionex Summit P680A HPLC system equipped with an ASI-100 auto sampler and a PDA-100 photodiode array detector. Glucosinolates were separated on a narrow bore 5 µm column (RP18 Acclaim ™ 120, 250×2.1 mm, Dionex) at a flow rate of 0.4 ml min^−1^ and a column temperature of 25°C. A 43 min gradient program was used with the eluents ultrapure water and 40% (v/v) acetonitrile (HPLC grade). Glucosinolate peaks were monitored at 229 nm. Glucosinolates were identified using standards, retention time, and UV spectra (see Table S1). The glucosinolate amount was calculated from the HPLC peak areas using response factors from desulfo glucosinolate at 229 nm.

Peak identity of the 13 desulfo GS detected in extracts was confirmed with LC-MS analysis on a HPLC-1100 series chromatograph (Agilent Technologies, Böblingen, Germany) coupled to an Esquire 6000 ESI-ion trap mass spectrometer (Bruker Daltonics, Bremen, Germany) operated in negative ion mode in the range m/*z* 50–700 as described in Mewis et al. (39). Following mass spectra of protonated molecular ions, [M+H]^+^ were obtained at certain retention times: 1) 3-methylsulfinylpropyl GS, m/*z* 344 and 183, 7 min, 2) 4-methylsulfinylbutyl GS, m/*z* 358 and m/*z* 197, 10 min, 3) 5-methylsulfinylpentyl GS, m/*z* 372 and 211, 11 min, 4) 6-methysulfinylhexyl GS, m/*z* 386 and 225, 13 min, 5) 4-hydroxy-indol-3-ylmethyl GS, m/*z* 385 and 224, 14 min, 6) 7-methylsulfinylheptyl GS m/*z* 400 and 239, 18 min, 7) 4-methylthiobutyl GS, m/*z* 342 and 181, 19 min, 8) 8-methylsulfinyloctyl GS, m/*z* 414 and 253, 20 min 9) indol-3-ylmethyl GS, m/*z* 369 and 207, 21 min, 10) 4-methoxy-indol-3-ylmethyl GS, m/*z* 399 and 237, 23 min, 11) 1-methoxy-indol-3-ylmethyl GS, m/*z* 399 and 237, 27 min, 12) 7-methylthioheptyl GS, m/*z* 344 and 223, 30 min, and 13) 8-methylthiooctyl GS m/*z* 398 and 237, 32 min.

For the analysis of glucosinolate in phloem sap, twenty phloem sap samples were pooled for each replicate. Analysis was carried out in five replications. Phloem sap samples were loaded directly on to the DEAE Sephadex A-25 mini columns for desulfation of glucosinolates as described above. Desulfated glucosinolates were analyzed by HPLC.

### Camalexin Quantification

Homogenized plant material, 20 mg, was extracted twice with methanol/ultrapure water (4/1, v/v) for 30 min at 65°C. Samples were analyzed by reverse phase HPLC using a RP-18 LiChroCART column (4×250 mm, 5 µm, Merck) and at a flow of 1 ml·min^−1^. The gradient program started with methanol/ultrapure water at 1∶1 for 2 min, followed by a 10 min linear gradient to 100% methanol and holding for 2 min at 100%, before returning to the initial run conditions. The peak at 10.2 min was identified as camalexin by comparison with verified standards with respect to retention time and UV spectrum (photodiode array detector PDA-100, Dionex) and quantified using a Shimadzu F-10AXL fluorescence detector (318 nm excitation, 370 nm emission).

### Chemical Analysis of Flavonoids

Flavonoids in *A. thaliana* samples were analyzed as aglycones. A 20 mg freeze-dried, ground sample was dissolved in 750 µl 80% (v/v) methanol (pH = 4) and sonicated at a temperature of 4°C for 20 min. After 5 min centrifugation at 4000 g, the extract was collected and the pellet re-extracted twice. Combined supernatants were concentrated in a centrifugation evaporator (Speed Vac, SC 110) to 250 µl and then an equal volume of 2M HCl was added. The extract was heated at 70°C for two hours in a thermo mixer at 400 rpm (Eppendorf, Germany) to hydrolyze the flavonoid glucosides to aglycones. The extract was cooled down and 100% methanol (HPLC grade) was added to the sample to a final volume of 1 ml. The extract was filtered through Spin-X centrifuge tube filters (Sigma-Aldrich, Inc.) and the flow-through was transferred to HPLC vials.

Samples were run on a Dionex Summit P680A HPLC system, equipped with an ASI-100 auto sampler and a PDA-100 photodiode array detector. The aglycones of flavonoids were separated on a narrow bore 3 µm column (AcclaimPA C16, 2.1×150 mm, Dionex) at a flow rate of 0.4 ml min^−1^ and a column temperature of 25°C. A 32 min gradient program was used with the eluents ultrapure water (pH 3, with acetic acid) and 100% acetonitrile (HPLC grade). Flavonoid aglycone peaks were monitored at 370 nm. Flavonoids were identified using standards, retention time, and UV spectra. The quantity of flavonoids was calculated from HPLC peak areas using the calibration curve from aglycone standards at 370 nm.

### RNA Isolation, cDNA Synthesis and Semi-quantitative RT-PCR Analysis

Total RNA was isolated from 100 mg of frozen *A. thaliana* rosettes with the RNeasy Plant Mini Kit (Qiagen GmbH, Germany) following the standard protocol. RNA was quantified by UV spectroscopy (Nanodrop ND1000, Technology Inc., USA) and its integrity was visually assessed on ethidium bromide stained agarose gels. RNA was first converted to cDNA using the Moloney murine leukemia virus reverse transcriptase according to the Promega protocol (Promega Corporation, USA).

Gene-specific primers were designed for PCR (Table S2) and the PCR reactions were optimized to ensure that the reaction was in the linear range for quantification by diluting the template. The PCR reaction was performed in a total volume of 20 µl, containing 5XGoTaq PCR buffer 4 µl (Promega), 0.2 mM dNTPs, 2.1 mM MgCl_2_, 0.5 µmol of the forward and reverse primer, 0.1 µl *Taq* DNA polymerase (Promega), and 1.5 µl of diluted cDNA. Then the PCR program was run on a thermo cycler (Applied Biosystems): 2 min at 96°C for initial denaturation, 30 cycles of 0.5 min at 94°C for denaturation, 0.5 min at 54°C for annealing, and 0.5 min at 72°C for extension, followed by a final extension for 7 min at 72°C. *Actin8* (AC8) was used as a reference gene. The forward (f) primer for AC8 was 5′-ATGAAGATTAAGGTCGTGGCAC and the reverse (r) primer 5′-GTTTTTATCCGAGTTTGAAGAGGC. To quantify the expression profiles, PCR products were run in gel electrophoresis (1.5% agarose gel containing SYBR® Safe DNA gel stain) and band intensities were visualized on a Biostep trans-illuminator. Phoretix Total Lab Quant software was used to measure band intensities and calculate the amount of PCR product using the low mass ladder (Invitrogen) for calibration. Expression of genes was normalized by dividing the band intensities with the corresponding *Actin8* intensities. The average gene expression levels in drought and water-logged treatments were compared to well-watered treatments, and gene expression levels in insect treatments were related to their controls in each water treatment to estimate the fold changes in transcript levels. The PCR analysis was carry out in 2 technical and 3 biological replications for each gene. We have also included drought stress marker genes such as *P5CS1* (DELTA1-PYRROLINE-5-CARBOXYLATE SYNTHASE 1), *PP2CA* (PROTEIN PHOSPHATASE 2CA), and *CDSP32* (CHLOROPLASTIC DROUGHT-INDUCED STRESS PROTEIN OF 32 KD), which have been shown to increase in microarray analysis (40) and also increased in response to drought stress in our analysis.

All primers successfully amplified a band of the correct size and PCR products were cloned into the T-overhang vector pCR2.1 with the TOPO TA cloning kit (Invitrogen). PCR products were fully sequenced to confirm their accuracy.

## Results

### Effects of the Water Status of Plants on Aphid Performance

**Figure 2:**
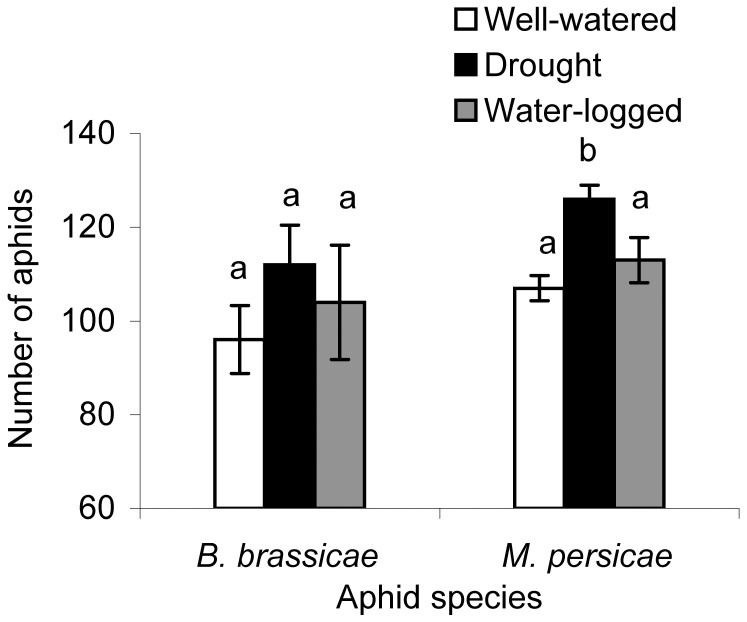
Drought stress favors the population growth of *Myzus persicae* on *A. thaliana*. Average population size of *B. brassicae* and *M. persicae* after feeding for 7 days on *A. thaliana* plants cultivated under different water conditions (n = 10, error bars: SE, different letters indicate significant differences in aphid species numbers between treatments, Tukey’s HSD test *P*<0.05).

Populations of the specialist *B. brassicae* increased between six and seven-fold within one week on *A. thaliana* Col-0 plants cultivated under well-watered, drought, and water-logged conditions, with no significant difference in population size between treatments (Fig. 2). In contrast, aphid numbers of the generalist *M. persicae* on *A. thaliana* plants differed between water treatments, with significantly higher numbers found on drought-stressed plants (Fig. 2).

### Effects of Water Availability on Plant Health and Metabolites: Plant Growth

Plant’s access to water can influence plant growth and the metabolite composition of phloem sap, which are important for the plant’s tolerance against aphids. We therefore investigated the relevant metabolites and plant parameters that could influence aphid infestation levels.

### Plant Growth

**Figure 3:**
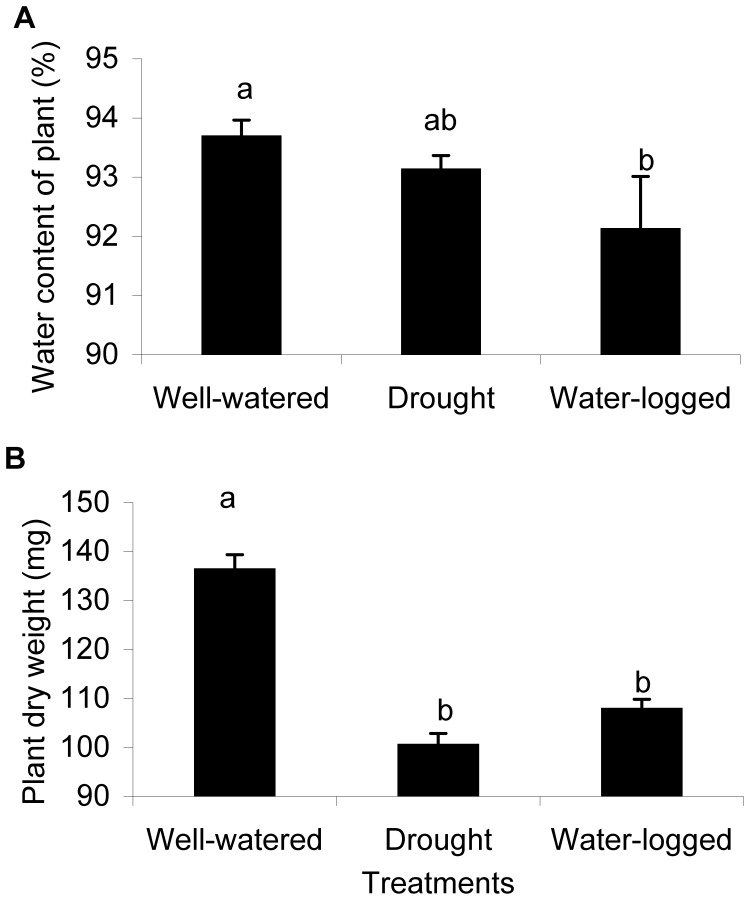
Water stress reduced plant water content and dry weight. Mean water content (A) and final dry weight (B) of *A. thaliana* plants after cultivation under different water conditions (n = 10, error bars: SD, different letters indicate significant differences in values obtained between treatments, Tukey’s HSD test *P*<0.05).

The water content of *A. thaliana* plants depended on the water status of the soil. Water content decreased under both drought and water-logged conditions, although significantly lower water content levels were determined for water-logged plants compared to well-watered plants (Fig. 3A). Also plant growth of *A. thaliana* was affected by water stress. Biomass production measured as dry weight was significantly lower in plants grown under drought and water-logged conditions than in well-watered plants (Fig. 3B).

### Free Amino Acids Levels in Phloem Sap

Eighteen different amino acids were detected in the phloem exudates of *A. thaliana* plants, showing that non-essential amino acids such as aspartic acid, serine, asparagines, glutamic acid, alanine, and proline dominate (Table 1). The concentration of amino acids in the phloem sap of plants that had experienced different water treatments varied significantly after one and two weeks of stress application. Total amino acid levels were higher in drought-stressed plants and lower in water-logged plants compared to well-watered plants; however, only the differences between amino acid concentrations in phloem sap of drought-stressed and water-logged plants were significant. Of the non-essential amino acids, particularly proline, glutamic acid, and aspartic acid were significantly higher in the phloem sap of drought-treated *A. thaliana* plants compared to water-logged plants (Table 1). Among the essential amino acids, concentrations of arginine, isoleucine, leucine, and lysine, were influenced the most by water availability, with levels increasing under drought stress and partially decreasing under water-logged conditions.

**Table 1:** Concentrations of amino acid in the phloem sap of *A. thaliana* plants cultivated under different water stress treatments.

Amino acid	Concentrations of amino acid in phloem sap (µmol g^−1^ dry weight)					
	1 week of water stress			2 weeks of water stress		
	Well-watered	Drought	Water-log	Well-watered	Drought	Water-log
***Essential*** *						
Arginine	0.731ab	1.065a	0.425b	0.477a	0.819a	0.551a
Histidine	0.356a	0.368a	0.359a	0.200a	0.332a	0.651a
Isoleucine	0.713ab	1.088a	0.349b	0.488ab	0.750a	0.343b
Leucine	1.061a	1.256a	0.509b	0.591a	0.987a	0.628a
Lysine	0.989ab	1.296a	0.646b	0.375a	0.928a	0.671a
Methionine	0.163a	0.223a	0.125a	0.157a	0.128a	0.380a
Phenylalanine	0.622a	0.788a	0.302a	0.430a	0.576a	0.471a
Threonine	2.863a	2.961a	2.218a	1.938a	2.282a	1.681a
Valine	1.241a	1.267a	0.776a	0.566a	0.997a	0.831a
***Non-essential***						
Alanine	5.058a	5.539a	4.532a	2.669a	3.313a	2.064a
Asparagine	4.688a	4.320a	4.978a	2.347a	2.342a	1.891a
Aspartic acid	6.671ab	8.077a	4.262b	3.772ab	6.585a	3.401b
Glutamic acid	4.837ab	7.262a	3.664b	3.379b	7.622a	3.154b
Glycine	0.587a	0.581a	1.007a	0.313a	0.319a	0.236a
Proline	2.843b	6.030a	1.474b	0.964b	5.710a	0.480b
Serine	3.229a	3.133a	3.139a	1.944a	2.392a	1.987a
Tyrosine	0.281a	0.271a	0.129b	0.139a	0.105a	0.137a
**Total**	**36.93ab**	**45.42a**	**28.89b**	**20.74ab**	**36.18a**	**19.21b**

n = 4, Mean values followed by different letters within a row and for one sampling day indicate significant differences for treatments, Tukey’s HSD test *P*<0.05;

* Essential amino acids as defined by Morris (1991).

### Sugar Levels in Phloem Sap

**Figure 4:**
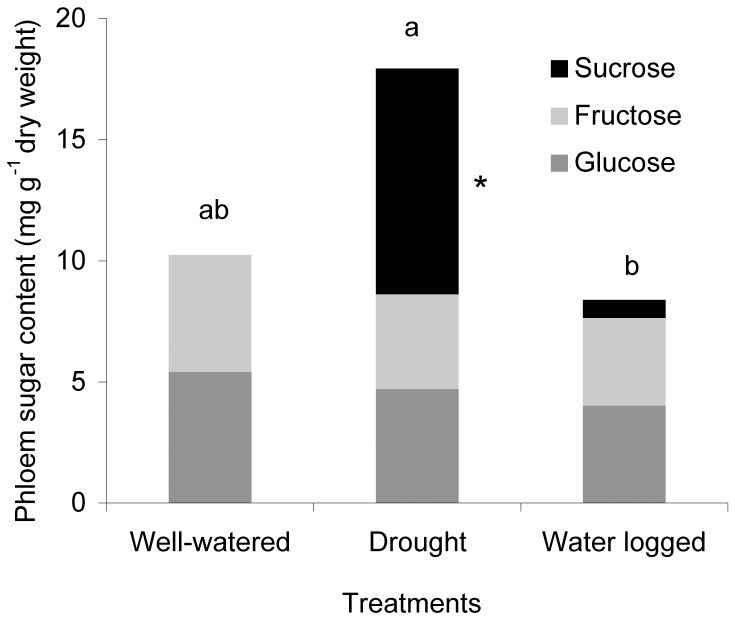
Sucrose levels in phloem sap increased under drought stress. Sugar levels in the phloem sap of *A. thaliana* plants cultivated under different water conditions (n = 3, different letters indicate significant differences between treatments, * indicates significant differences among treatments of sucrose only, Tukey’s HSD test *P*<0.05).

After one week, the sugars levels (glucose, fructose, and sucrose) in the phloem sap of *A. thaliana* plants were not influenced by the different water stress treatments. However, after continued cultivation under water stress, significantly higher amounts of sucrose were detected in the phloem sap of drought-stressed plants compared to well-watered and water-logged plants, which both had little or no detectable amounts of this carbohydrate (Fig. 4).

### Glucosinolates in Phloem Sap

**Figure 5:**
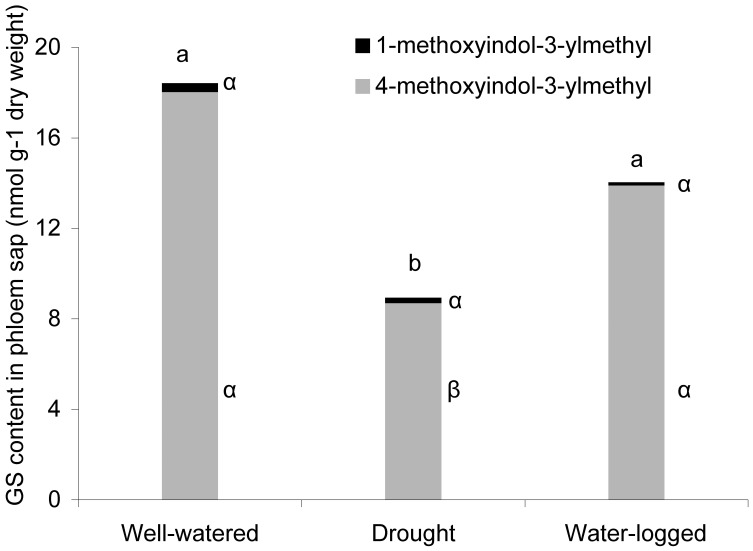
Drought stress reduced indolyl glucosinolate concentrations in the phloem sap of plants. Glucosinolate (GS) levels in the phloem sap of *A. thaliana* plants cultivated for one week under different water conditions (n = 3, different letters indicate significant differences in total glucosinolate levels between water treatments, and different Greek letters indicate significant differences in single compounds between the water treatments, Tukey’s HSD test *P*<0.05).

Only two glucosinolates (GS) were detected in the phloem sap of mature *A. thaliana* plants, namely 1-methoxyindol-3-ylmethyl and 4-methoxyindol-3-ylmethyl glucosinolate. These levels did not change significantly within one week of water stress (ANOVA: F_2,11_ = 1.3, *P* = 0.337). But as the period of stress continued, 4-methoxyindol-3-ylmethyl glucosinolate concentrations decreased significantly in drought-stressed plants (Fig. 5, Tukey’s HSD test *P*<0.05).

### Correlation Results of Aphid Performance to Compounds in Phloem Sap

Simple correlations were used to explore the likelihood that constitutive metabolite levels in the phloem sap of plants under different water conditions might be a basis of plant suitability for aphid species. The number of aphids and increasing indolyl glucosinolate concentrations in the phloem sap were inversely related, whereby the correlation coefficient was stronger for *M. persicae* with R = −0.802 than for *B. brassicae* with R = −0.617. In contrast aphid performance was positive related to primary metabolite content in the phloem sap. *M. persicae* population increased with a somewhat stronger positive correlation to carbohydrate and amino acid concentrations in the phloem sap, with R = 0.718 and R = 0.754 compared to *B. brassicae* with R = 0.613 and R = 0.542.

### Effects of Water Availability on Plant Secondary Metabolites and the Response to Aphid Feeding

Besides analyzing water stress-induced changes in compounds found in phloem sap, which directly influences phloem feeding insects, secondary metabolite accumulation in water-stressed plants was investigated to elucidate changes within the general plant defense response.

### Glucosinolate Response

Thirteen different glucosinolates were identified in mature *A. thaliana* Col-0 rosettes (Table S1). Levels of aliphatic glucosinolates, but not indolyl glucosinolates, changed significantly upon water stress (ANOVA: F_2,14_ = 5.55, *P* = 0.021). Higher aliphatic glucosinolate amounts were determined in drought-stressed plants compared to well-watered plants, with intermediate levels in water-logged plants. Significant increases in 4-methylsulfinylbutyl and 5-methylsulfinylpentyl glucosinolate concentrations were responsible for an increase in total aliphatic glucosinolate levels in drought stressed plants (Tukey’s HSD test *P*<0.05). Furthermore, 4-methoxyindol-3-ylmethyl glucosinolate significantly decreased under drought stress conditions Tukey’s HSD test *P*<0.05.

**Figure 6:**
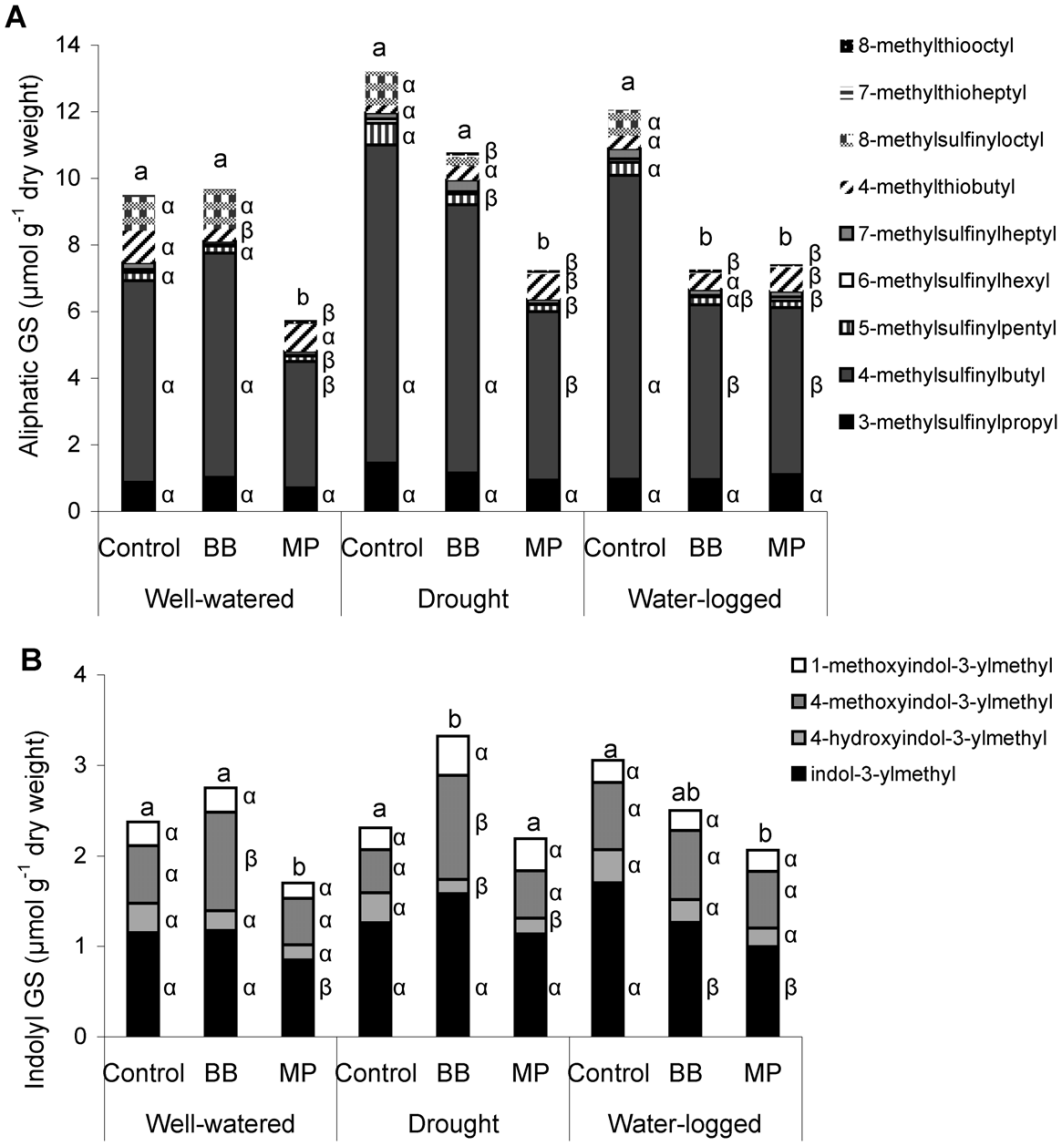
Especially *M. persicae* reduced aliphatic glucosinolate levels under all water conditions. Glucosinolate (GS) levels in *A. thaliana* plants cultivated under different water conditions and after one week feeding of *M. persicae* (Mp) and *B. brassicae* (Bb) (n = 5, different letters indicate significant differences in total glucosinolate levels of control and aphid-treated plants within a water treatment, different Greek letters indicate significant differences in single compounds within each water treatments for the main compounds, Tukey’s HSD test *P*<0.05; minor compounds not significantly different).

Feeding of aphids changed the glucosinolate profile of *A. thaliana* after one week (Fig. 6). The induced plant changes were aphid species-specific, and especially feeding of *M. persicae* lead to significantly decreased aliphatic glucosinolates in all water treatments (Fig. 6A). Contrary to the generalist aphid, feeding by the specialist *B. brassicae* reduced aliphatic glucosinolates significantly only in water-logged plants (Fig. 6A). In response to aphid feeding especially the portion of methylsulfnylalkyl glucosinolates decreased. Indolyl glucosinolates in plants decreased only under well-watered and water-logged conditions in response to feeding of *M. persicae* (Fig. 6B). Furthermore, under drought conditions feeding by the specialist *B. brassicae* significantly induced levels of indolyl glucosinolates, especially of 4-methoxyindol-3-ylmethyl glucosinolate (Fig. 6B).

### Camalexin Response

**Figure 7:**
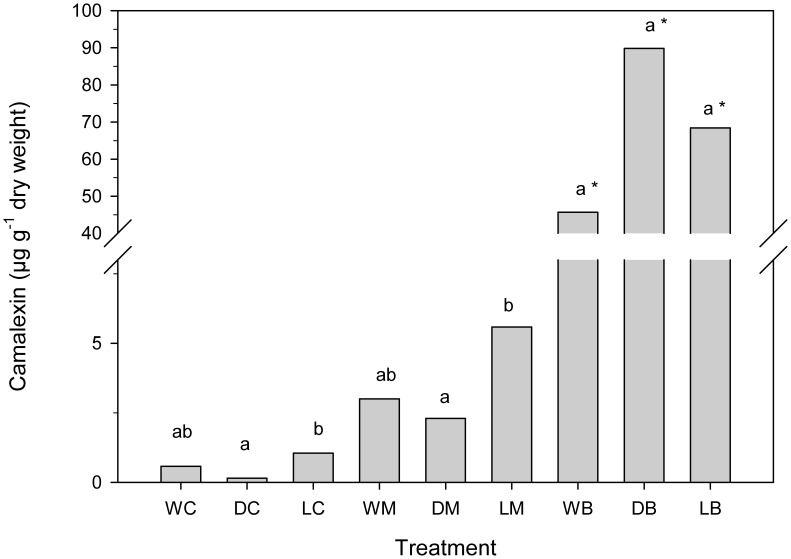
The aphid species elicited different camelexin accumulation in plants under different water treatments. Camalexin levels in control and aphid infested *A. thaliana* plants after two weeks cultivation under different water conditions (n = 5, different letters indicate significant differences between water treatments and bars with an asterisk indicate significant differences in *B. brassicae* infestation compared to *M. persicae* and controls, Tukey’s HSD test, *P*<0.05; WC: well-watered control, DC: drought control, LC: water-logged control, WM: well-watered + *M. persicae*, DM: drought + *M. persicae*, LM: water-logged + *M. persicae*, WB: well-watered + *B. brassicae*, DB: drought + *B. brassicae*, LB: water-logged + *B. brassicae*).

Water stress altered levels of camalexin in *A. thaliana* plants (Fig. 7). Camalexin content was determined as being twice as high in water-logged plants compared to well-watered plants, approximately four-fold less in plants under drought stressed conditions. However, camalexin levels were only significant different between water-logged and drought-stressed plants (Fig. 7). A similar pattern was obtained under infestation with *M. persicae,* albeit camalexin levels in general were slightly higher. Interestingly, camalexin was induced up to 90-fold in *A. thaliana* after feeding by *B brassicae*, regardless of the plant’s water availability.

### Flavonoid Response

**Figure 8:**
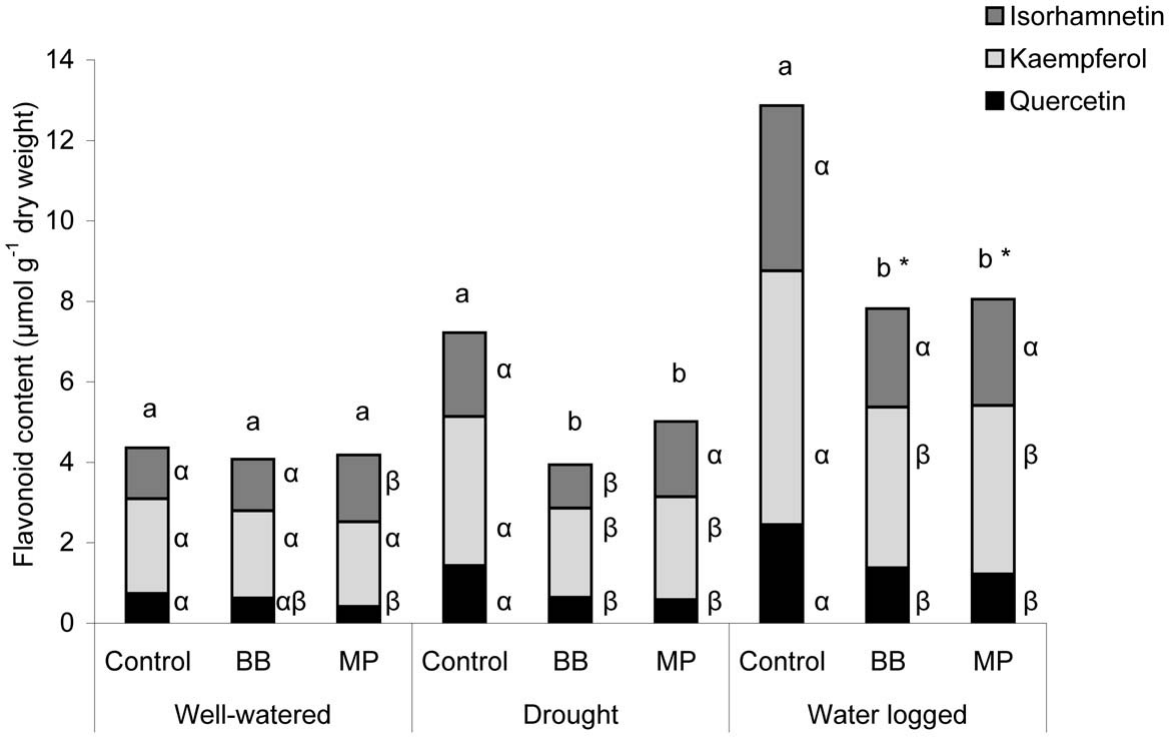
Flavonoid levels are influenced by aphids and water stress. Flavonoid content of *A. thaliana* after two weeks under different water conditions and one week of infestation with *M. persicae* (MP) and *B. brassicae* (BB) compared to non-insect treated plants (n = 5, different lower case letters indicate significant differences in total flavonoid content within the water treatments; different Greek letters indicate significant differences in single compounds within the water treatments and listed on the right; Bars with an asterisk indicate differences within the flavonoid content of water-logged plants compared to respective treatments on well-watered and drought-stress plants for single and total levels; Tukey’s HSD Test *P*<0.05).

Mature *A. thaliana* plants contained isorhamnetin, kaempferol, and quercetin as flavonoids (Fig. 8). Level of flavonoids were not significantly different in the plants after one week of treatment (one way ANOVA, F_2,14_ = 1.7, *P* = 0.222). However, after two weeks of water stress, significantly higher levels of flavonoids were detected in water-logged plants, followed by drought-stressed plants, when compared to well-watered plants (Tukey’s HSD test *P*<0.05). Aphid feeding did not elicit overall changes in the flavonoid content of plants under well-watered conditions. In contrast, plants under drought, and particularly water-logged stress conditions had overall higher levels of flavonoids, which after feeding of both aphid species decreased substantially in (Fig. 8.) Among the above-mentioned flavonoids, only quercetin concentrations decreased significantly following feeding of either aphid under all the water treatments.

### Water Stress and Aphid-mediated Changes in Glucosinolate Biosynthetic and Regulatory Gene Transcript Levels

Since glucosinolate levels of *A. thaliana* were influenced by water treatment and aphid feeding, we analyzed the expression of selected genes associated with glucosinolate biosynthesis and signaling pathways. Under drought stress, *MAM1* and two transcription factors involved in aliphatic glucosinolate biosynthesis - *MYB29* and *MYB76* - were up-regulated two-fold, while genes related to indolyl GS remained unchanged, except for *CYP79B2*, also involved in camalexin biosynthesis, which decreased (Table 2). Also the transcript level of *PAD3* responsible for the final step in camalexin biosynthesis, decreased about two-fold. Under water-logged conditions, only *MYB76* transcription increased. From genes associated with signaling pathways, only the expression level of *PR1*, which is associated with the salicylic acid pathway, decreased under drought stress.

**Table 2:** Transcript levels of glucosinolate biosynthetic, regulatory and camalexin biosynthesis genes in *A. thaliana* after two weeks’ water stress and one week of aphid feeding (plants39 days old, n = 3 biological replicates).

	Mean change in transcript level							
	Water treatment		Induction by aphid species in water treatments					
			Well-watered		Drought		Water-logged	
	Drought	Water-logged	*M. persicae*	*B. brassicae*	*M. persicae*	*B. brassicae*	*M. persicae*	*B. brassicae*
**Glucosinolate biosynthesis genes**								
*MAM1*	0.81	0.25	−2.28*	−3.81*	−2.70*	−1.59*	−1.74	−0.84
*MAM3*	−0.13	−0.45	−0.41	−0.30	0.30	−0.28	−0.34	−0.28
*CYP79F1*	0.23	0.20	−22.50*	−140.69*	−70.31*	−128.55*	−191.06*	−51.96*
*CYP 83A1*	0.08	0.58	−0.90**	−0.96**	−2.00*	−0.80	−2.20*	−0.68
*UGT74C1*	−0.02	0.07	−5.56	−3.76	−5.23	−4.55	−6.01	−2.47
*SUR1*	0.08	0.07	−0.18	−0.39	−0.44	−0.04	−0.13	0.11
*CYP79B2*	−1.82	−0.51	−0.42	0.19	0.35	3.24	0.32	1.92*
*CYP79B3*	0.22	−0.51	−0.18	0.84	0.16	1.35	0.83	1.97
*CYP83B1*	0.05	0.54	0.19	0.53	0.18	0.60	−0.16	0.19
*UGT74B1*	−0.22	−0.16	−1.03	−0.41	−1.22	−0.12	−0.99	−0.11
*MYB28*	0.47	−0.19	−2.49**	−3.30**	−5.11*	−2.83*	−1.10	−0.38
*MYB29*	1.04	0.25	−1.58	−1.96	−4.87*	−5.11*	−6.86**	−4.19**
*MYB76*	1.61	1.31	−0.13	−0.10	−1.14**	−1.69**	−0.80	−0.83
*MYB34*	0.19	0.07	−1.28	−0.87	−3.82*	−4.52*	−0.70**	−1.13*
*MYB51*	−0.04	−0.24	1.14	0.61	1.02	1.56	1.91*	2.63*
*MYB122*	−0.13	−0.07	−0.22	1.68*	0.43	2.99*	0.56	3.48*
*TGG1*	−0.21	−0.08	−0.72**	−0.77**	−0.48**	−0.42	−0.64	−0.91
**Signaling pathway genes**								
*PR1*	−1.55	−0.06	−0.16	0.32	0.72	1.54	0.01	0.28
*CaEF*	−0.40	−0.26	−0.04	−0.24	−0.19	−0.17	0.03	−0.12
*BGL1*	−0.19	0.10	−1.33*	−2.21*	−0.89	−0.31	−0.50**	−0.47**
*PAD3*	−0.70	0.27	2.62	4.93*	3.59	10.33*	7.83*	11.13*

Positive value: fold increase, negative value fold decrease, *and **indicate significant differences in transcript levels at *P*<0.1 and *P*<0.05, Tukey’s HSD test.

Although aliphatic glucosinolates decreased in *A. thaliana* only after feeding of *M. persicae* in all water treatments, transcript levels of biosynthetic genes *MAM1*, *CYP79F1*, *CYP83A1*, and *UGT74C1* decreased after infestation with either aphid species (Table 2). Feeding of *M. persicae* did not alter the expression of genes involved in earlier indolyl glucosinolate biosynthesis steps. Furthermore, expression of genes encoding aliphatic glucosinolate biosynthesis transcription factors, i.e. *MYB28, MYB29*, and *MYB76*, was mostly down-regulated by *M. persicae* and *B. brassicae* in both water stress treatments. Notably, expression of genes encoding indolyl and aliphatic glucosinolate biosynthesis transcription factors responded differentially to aphid feeding in *A. thaliana*, with increased *MYB51* and decreased *MYB76*. Transcript levels of *MYB122* were up-regulated as a result of feeding by *B. brassicae* in all water treatments.

Interestingly, infestation with *B. brassicae* increased transcripts of *CYP79B2* and *CYP79B3* in drought and water-logged plants, which corresponds to the accumulation of indolyl glucosinolates and camalexin in such challenged plants. Feeding of both, *M. persicae* and *B. brassicae*, up-regulated the expression of *PAD3* in *A. thaliana*, but induction was stronger in response to *B. brassicae*. One gene encoding for myrosinase1 in *A. thaliana*, *TGG1*, was included in our study. Water stress did not change its expression levels, but plant infestation with *M. persicae* or *B. brassicae* down-regulated *TGG1* in all treatments.

From the signaling pathway genes, aphid feeding did not change expression levels of *CaEF*, a gene that responds to ethylene (Table 2). *B. brassicae* up-regulated *PR1* only under drought stress, while the salicylic acid responsive gene remained unchanged upon aphid feeding in both well-watered and water-logged conditions. *BGL1*, commonly induced by JA, was down-regulated after feeding by either *M. persicae* or *B. brassicae* in all water treatments.

**Figure 9:**
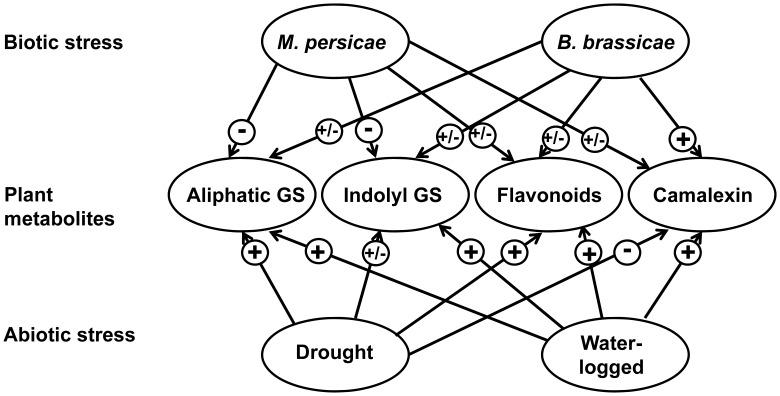
Overall effects of aphid species and water stress on secondary metabolite accumulation in *A. thaliana*. Scheme depicting the effect of aphid infestation (biotic stress) or water stress (abiotic stress) on inducing (+), reduction (−), or not changing (+/−) plant metabolites.

## Discussion

### Water Mediated Effects on Metabolite Composition in Phloem Sap Promoted Only Growth of Myzus Persicae

Aphids feed exclusively on phloem sap and their association with plants is believed to depend on the quality (metabolite composition) of the sap. Besides the negative effects of water stress on plant growth, the present study revealed distinct changes within the chemical metabolite composition in the phloem sap. Drought stress enriched the nutritional quality of phloem sap in *A. thaliana* Col-0 plants by increasing the amounts of free amino acids and sugars (Table 1 and Fig. 4). Several plant families, including Brassicaceae, translocate sucrose or raffinose together with one polyol in the phloem sap for long-distance allocation of photo-synthetically fixed carbon [40]. As efficient osmolytes, many plant species accumulate polyols under salt or drought stress. This is in agreement with the remarkable accumulation of sucrose in the phloem sap of drought-stressed *A. thaliana* plants observed in this study. It is also hypothesized that elevated free amino acids act as osmolytes, protecting cells from dehydration. Correspondingly, several essential amino acids (e.g. leucine and isoleucine) and non-essential amino acids (e.g. proline and glutamic acid) increased in plants following drought stress in our study, but declined under water-logged conditions in *A. thaliana*. Proline, one of the known markers for water stress, increased dramatically in drought stressed plants when compared to their respective controls, whereby proline has been shown to increase in several different plant species and is thought to provide an osmoprotective function [41], [42]. Accumulation of free isoleucine and other branched-chain amino acids in response to drought stress was also reported by Joshi and Jander [43].

Higher sugar content in their diet, especially sucrose, has been shown to increase the performance of aphids [44], and we found a positive correlation of the two aphid species to increasing sugar levels in the phloem sap of plants under different water condition. However, in other studies high sugar content in the sap had no effect on aphids or was considered as barrier to aphid feeding [45]. Also amino acid concentrations, e.g. tyrosine, alanine, leucine, and glutamic acid, in *Brassica* species were reported to account for 43% of variations in the intrinsic rate of increasing populations of the generalist *M. persicae* and the specialist *B. brassicae* [46]. However, the supply of essential amino acids in phloem sap may be comparatively less important for aphid performance, since they are able to obtain these amino acids from their symbiotic bacteria, such as *Buchnera aphidicola*, in exchange for providing the bacteria with non-essential amino acids, e.g. aspartic/glutamic acid, as well as sucrose [45], [47]. Our observed greater population growth of *M. persicae* on drought stressed *A. thaliana* compared to well-watered and water-logged plants is in line with the enriched quality (increased amino acids) of phloem sap in water deficient plants (Fig. 2, Fig. 4, and Table 1). Karley et al. [48] reported that *M. persicae* and *Macrosiphum euphorbiae* (Thomas) perform best on young potato plants and with an artificial diet enriched with non-essential amino acids, particularly glutamine and asparagine, which is consistent with our findings. That increasing amino acids in phloem sap is beneficial for *M. persicae* is also confirmed in the present study, since a positive correlation of aphid population growth to increasing amino acid levels in differently water treated plant was determined. However, the performance of the specialist aphid, *B. brassicae* was not influenced by the plant’s water availability. Drought stress can make the plant more or less suitable for attacking herbivores, depending on drought intensity and insect species. In fact, there was only a weak relationship of *B. brassicae* population growth to increasingg amino acid levels indicating other metabolites make drought stress plants less suitable. Water deficiency in *Brassica napus* also negatively influenced *B. brassicae* and had no effect on the population growth of *Lipaphis erysimi* (Kaltenbach) [10].

Secondary metabolites present in the phloem sap can also alter host-plant suitability for aphids and could contribute to the observed different effects of drought stress on aphid performance. The concentration of glucosinolates in the phloem sap of *A. thaliana* differed between the various water treatments, and we determined lower amounts of 4-methoxyindol-3-ylmethyl glucosinolate under drought stress than in well-watered and water-logged plants (Fig. 5). Using artificial diets it was previously shown that indolyl glucosinolate breakdown products have an anti-feeding effect on *M. persicae* and reduced aphids’ reproduction [12]. The same authors showed that *atr1D* mutant plants overproducing indolyl glucosinolates are more resistant to *M. persicae*, whereas *cyp79B2cyp79B3* double mutants lacking indolyl glucosinolates are more suitable to *M. persicae* growth. A stronger effect of 4-methoxyindol-3-ylmethyl glucosinolate compared to indole-3-ylmethyl glucosinolate was determined for *M. persicae* in the absence of myrosinase [16]. Our observed lower amount of 4-methoxyindol-3-ylmethyl glucosinolate in the phloem sap of drought-stressed plants, together with higher amino acid and sucrose content, may cause the observed improvement in population growth of *M. persicae* (Fig. 2). However, glucosinolate levels may not explain the performance of *B. brassicae* on plants receiving the water stress treatments. In contrast to *M. persicae*, this specialist can tolerate glucosinolates to some extent and sequesters these compounds to protect itself against enemies [49], [50]. The improvement in phloem sap quality in drought stressed plants had little effect on *B. brassicae*, indicating a possible alteration in further growth relevant metabolites. Equivalent performance of *B. brassicae* on broccoli plants cultivated under different water conditions has been reported by Khan et al. [2], [3]. High glucosinolate concentrations in the well-watered and water-logged plants may have influenced the feeding habits of *B. brassicae*, causing them to ingest more phloem sap, as reported by Gabrys et al. [51]. Consequently, they would ingest amounts of nutrients similar to normal feeding on drought-stressed plants.

### Water Stress Alters Plant Defense Metabolites and Response to Aphids

Different water availability may change both the plant’s constitutive levels of secondary metabolites and its induced defense response to herbivory. Plant growth and defenses are restricted by their internal resources and according to the growth-differentiation balance hypothesis, plants must strike a balance between growth processes and production of defensive compounds [52]. Considering these relationships, low stress conditions with unlimited growth will result in no increase in secondary metabolites, whereas moderate water stress may result in elevating secondary metabolites; however, secondary metabolites should decrease under strong and significant water limitation.

With this hypothesis in mind, we found reduced growth in plants under both water stress conditions along with increased levels of aliphatic glucosinolates and flavonoids under drought stress; flavonoids increased most under water-logged conditions (Fig. 3 and Fig. 8). Similarly, elevated levels of flavonoids under drought and water-logged conditions were observed for broccoli plants [3]. In several studies, a drought-mediated glucosinolate elevating effect was determined for *Brassica* species [53], [54], [55]. However, glucosinolate accumulation was shown to depend on drought intensity, and glucosinolate levels remained unchanged [52] or decreased under drought conditions in some species [3], [56]. Changes in glucosinolate levels in the plant tissue should influence chewing insects, as reported by Mewis et al. [57] and Gutbrodt et al. [56], but may not affect aphids which feed on phloem sap.

It has been proposed that glucosinolates are produced in drought tissue at low turgor and that under these conditions glucosinolate precursors are produced for later use [53]. Indeed, we noticed a trend towards increasing levels of branch chain amino acids, including methionine, in the phloem sap of *A. thaliana* under drought stress, but levels of 4-methoxy-indol-3ylmethyl glucosinolate decreased. Along with increased aliphatic glucosinolates in drought-stressed plants, transcript levels of *MAM1* and two transcription factors involved in aliphatic glucosinolate biosynthesis, *MYB29* and *MYB76*, increased. *MYB29* and *MYB76* over-expression was reported to noticeably increase levels of 3-methylsulfinylpropyl, 4-methylsulfinylbutyl, and 5-methylsulfinylpentyl glucosinolate [24]. MAM1 catalyzes only the short chain methionine derivatives, resulting in accumulation of short-chained aliphatic glucosinolates [58]. Although *MYB76* was slightly up-regulated in water-logged plants, the glucosinolate profile of these plants did not change. MYB76 was shown to integrate different stress responses with aliphatic glucosinolate regulation, while MYB29 is more important for maintaining a basic level of aliphatic glucosinolates, not only in non-stress situations but also in response to environmental cues [24]. Interestingly, both drought and water-logged conditions had no overall-effect on indolyl glucosinolate accumulation and corresponding gene expression.

The two aphids induced partially different plant responses in the present study, and this was influenced by the water available to the plants. Aliphatic glucosinolate decreased after *M. persicae* feeding on *A. thaliana* in all water treatments, while feeding of *B. brassicae* only reduced levels of aliphatic glucosinolate in drought-stressed plants (Fig. 6A). A similar decrease in total aliphatic glucosinolate content in Col-0 plants in response to *M. persicae* feeding was observed by Kim et al. [12]. Contrary to their results, in our study *M. persicae* did not influence levels of 4-methoxyindol-3-ylmethyl glucosinolate in Col-0; however this indolyl defense compound accumulated in response to *B. brassicae* feeding (Fig. 6B). Decreased glucosinolate contents in *M. persicae-*infested, water-stressed plants were associated with down-regulation of aliphatic glucosinolate biosynthetic (*MAM1*, *CYP79F1*, *CYP83A1*, and *UGT74C1*) and transcription factor genes (*MYB28*, *MYB29*, and *MYB76*, Table 2). Response to *B. brassicae* only reduced aliphatic glucosinolates in water-logged plants; however biosynthetic core structure genes as early response were down-regulated in response to feeding under all water conditions. Kuśnierczyk et al. [34] reported that the transcript levels of genes associated with the aliphatic glucosinolate biosynthesis pathway were either unchanged or slightly down-regulated upon infestation by *B. brassicae* and *M. persicae*, but in their study, aphids fed on the plant for only three days and not for seven days, as in our study.

Interestingly, the generalist *M. persicae* and the specialist aphid *B*. *brassicae* modulated the transcript profile of indolyl glucosinolate biosynthetic genes differently. While the feeding of *M. persicae* induced no changes in transcripts involved in earlier steps of glucosinolate biosynthesis in *A. thaliana*, *B. brassicae* induced expression of *CYP79B2* and *CYP79B3* under drought and water-logged conditions, along with increased levels of 4-methoxyindol-3ylmetyl glucosinolate (Table 2). Furthermore, two of the three transcription factors which induce indolyl glucosinolate biosynthesis, *MYB51* and *MYB122* [22], were only up-regulated in differently water treated plants only in the case of *B. brassicae*. Unlike our study, Kuśnierczyk et al. [59] found increased expression indolyl glucosinolate biosynthesis genes such as *CYP79B2* and *CYP79B3* after a short feeding period of both *M. persicae* and *B. brassicae*.

*TGG1*, encoding for the enzyme myrosinase, was down-regulated after infestation by *M. persicae* and *B. brassicae* in plants subjected to all water conditions. This observation is consistent with the studies of Kuśnierczyk et al. [59], where both myrosinase encoding genes *TGG1* and *TGG2* are down-regulated after feeding of *B. brassicae* and *M. persicae*. Down-regulation of the myrosinase encoding genes may have been irrelevant for *M. persicae* and *B. brassicae*, since they can avoid the myrosinase-glucosinolate defense system of plants. Indeed, knocking out *TGG1* and *TGG2* did not influence the performance of *M. persicae* [60]. Rather, this change might increase the plants’ susceptibility to chewing insects and pathogens, since glucosinolate breakdown products have been shown to suppress microbial growth [15].

Feeding of aphids decreased flavonoid accumulation under drought and water-logged stress (Fig. 8). Flavonoids benefit the plant since they are scavengers of reactive oxygen species (ROSs) under stress conditions and can influence insect herbivory [61], [62], but maybe not aphid growth. Lattanzio et al. [63] and Kim et al. [64] suggested that elicitors released by aphids during feeding manipulate flavonoid resistance mechanisms.

Our study also provides evidence that camalexin accumulation depends on the water available to the plant. Transcript levels of the camalexin biosynthetic genes *CYP79B2* and *PAD3* were down-regulated by drought stress, resulting in less camalexin in plants under drought conditions than in plants under excessive water. The feeding of *B. brassicae*, but not *M. persicae*, increased expression of *CYP79B2*, *CYP79B3*, and *PAD3* resulting in a 50 to 100-fold increase in the concentration of camalexin, known as a phytoalexin active against pathogens, content in plants under all water conditions (Fig. 7). In comparison, the camalexin response elicited by the generalist *M. persicae* was marginal and statistically not significantly different from the corresponding water treatment controls. Consistent with our finding, a previous study by Kuśnierczyk *et al.* [34] found increased camalexin in *B. brassicae-*infested plants. However, their study only included one aphid species and no water stress.

In theory, the improved phloem sap quality in drought-stressed plants should also favor the specialist aphid, and not just the generalist aphid. Since the highest camalexin levels were found in drought-stressed, *B. brassicae-*infested *A. thaliana* plants one could speculate whether an alternative chemical defense mechanism exists against this specialist, reasonably glucosinolate-tolerant aphid. The aphid fitness experiment conducted by Kuśnierczyk et al. [34] found that *B. brassicae* was significantly more fecund on camalexin-lacking *pad3-1* mutants than on wild-type plants. This supports our hypothesis that high camalexin levels in drought-treated plants reduce their suitability as hosts. Further studies are required, however, to prove that camalexin is present in phloem sap. Interestingly, Pegadaraju et al. [65] observed no differences in population growth of *M. persicae* on *pad3-1* or wild-type plants. They concluded that camalexin has no effect on this generalist aphid. However, our study rather suggests that the similar performance of *M. persicae* on the two genotypes could be attributed to the very low induction of camalexin in Col-0 by this aphid species.

Genes associated with signaling pathways were included in this study to elucidate cross talk in plant responses to different stressors. In a drought assay using *A. thaliana* mutants modified in abscisic acid and jasmonate, Harb et al. [66] found that the associated network of these plant hormones is crucial for the plants’ response to drought. In our study, *BGL1* (responsive to jasmonic acid) and *CaEF* (ethylene signaling) expression did not change [67], but lower levels of *PR1* transcript indicate down-regulation of salicylic acid signaling (Table 1). The plants’ response to aphids is partially associated with the salicylic acid pathway, and *PR1* is induced by aphids, as reported by Moran and Thomson [17] and Mewis et al. [18]. In this study, *PR1* was only up-regulated by *B. brassicae* in drought-stressed plants, along with accumulation of 4-methoxyindol-3-ylmethyl glucosinolate. Exogenous application of salicylic acid has been shown to specifically promote the accumulation of 4-methoxyindol-3-ylmethyl glucosinolate in *A. thaliana* [67], [68]. With this in mind, higher salicylic acid levels in response to *B. brassicae* infestation could lead to the accumulation of 4-methoxyindol-3-ylmethyl glucosinolate in the plant. Drought treatment alone down-regulated the transcripts of *PR1* and significantly lowered 4-methoxyindol-3-ylmethyl glucosinolate contents in drought-stressed plants. This finding suggests that the salicylic acid signaling pathway is a key element in regulating 4-methoxyindol-3-ylmethyl glucosinolate production from the indol-3-ylmethyl glucosinolate precursor.

In addition to the importance of salicylic acid signaling, accumulation of glucosinolates is often linked to the jasmonic acid pathway. Exogenous application of methyl jasmonate and jasmonic acid was reported to facilitate the induction of glucosinolate biosynthesis genes, with corresponding increases of indolyl glucosinolates in *A. thaliana* [57], [68]. In our study, aphid feeding decreased transcripts of *BGL1* in plants under all conditions, while levels of CaEF remained unchanged. Corresponding to the down-regulation of jasmonic acid signaling, aliphatic and indolyl glucosinolates decreased after feeding of *M. persicae,* irrespective of the water treatment. The observed *B. brassicae* induced accumulation of indolyl glucosinolate under drought stress seems to be jasmonate-independent (Fig. 6B) and might be also one explanation for the similar performance on drought and well-water plants, which might be contrary to the initial lower constitutive levels of indolyl glucosinolates in the phloem sap.

### Conclusions

The drought stress-mediated enrichment of phloem sap with free amino acids, along with the decreased concentration of 4-methoxyindol-3-ylmethyl glucosinolate promoted population growth of the generalist aphid *M. persicae* on *A. thaliana*. The specialist *B. brassicae* did not particularly profit from the increased phloem sap quality, since it performed equally well on plants cultivated under different water conditions. Plants responded to infestation with *B. brassicae*, but not with *M. persicae*, by producing high levels of camalexin in plant tissues under all water conditions. This may lead to decreased host plant suitability for this aphid species. However, further investigations are required to determine possible transport, and, therefore, presence of camalexin in phloem sap.

Water stress and aphids generally influenced the accumulation of secondary metabolites an opposite ways and participated differentially in signaling pathways as summarized in Figure 9. Water-logging and drought stress led to increased aliphatic glucosinolate and flavonoid levels in the plants. Conversely, aphid feeding, especially of *M. persicae*, reduced the accumulation of glucosinolates and flavonoids in most water treatments, along with down-regulation of the jasmonic acid pathway. How the plants responded with secondary metabolite accumulation was obviously very different after feeding of *M. persicae* and *B. brassicae* and depended on the water status of the plants. For example, *B. brassicae* induced the salicylic acid pathway only under drought stress, meaning the plants accumulated 4-methoxyindol-3-ylmethyl glucosinolate. Different chemical elicitor present in the saliva might mediate the discriminative plants’ response to *M. persicae* and *B. brassicae*, a question that remains to be investigated in the future. Our findings suggest a complex network of regulating secondary metabolite biosynthesis in response to multiple environmental factors and a fine-tuned plant response to stressors, excluding a general prediction. Further experimentations should include GUS transformed *A. thaliana* mutants to visualize camalexin induction by the different aphid species. Also a direct comparison of *M. persicae* and *B. brassicae* performance on indolyl GS lacking *cyp79B2cyp79B3* mutants and camalexin deficient *pad3-1* mutants under different water conditions may be worth further investigation.

## Supporting Information

Table S1 UV-spectra of desulfo GS from *Arabidopsis* (Col-0).
(PDF)

Table S2 Primers list of genes associated with glucosinolate, camalexin biosynthesis or signaling pathways.
(PDF)
