# Supplementary material for: Water Stress and Aphid Feeding Differentially Influence Metabolite Composition in Arabidopsis thaliana (L.)
Source: PLoS One. 2012 Nov 7;7(11):e48661. doi: 10.1371/journal.pone.0048661 (PMC3492492; doi:10.1371/journal.pone.0048661)
Supplement: Table S1 — UV-spectra of desulfo GS from Arabidopsis (Col-0). (PDF) [file pone.0048661.s001.pdf]

Table S1: UV-spectra of desulfo GS from *Arabidopsis* (Col-0)

Glucosinolate (GS)	Spectrum
3-methylsulfinylpropyl GS	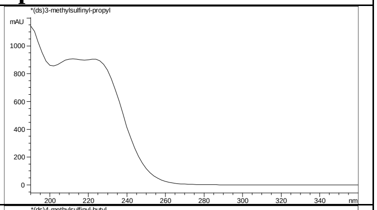
4-methylsulfinylbutyl GS	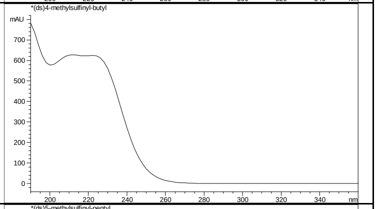
5-methylsulfinylpentyl GS	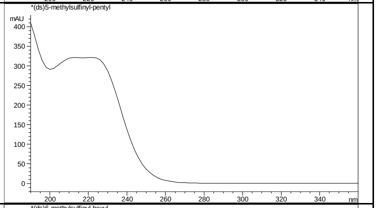
6-methylsulfinylhexyl GS	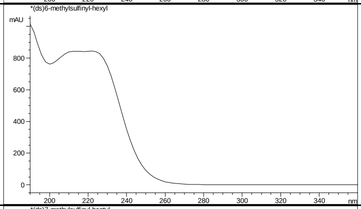
7-methylsulfinylheptyl GS	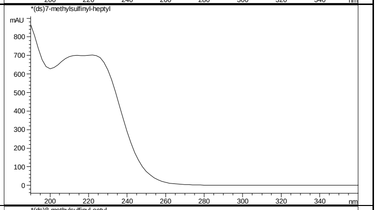
8-methylsulfinyloctyl GS	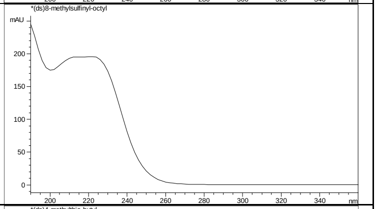
4-methylthiobutyl GS	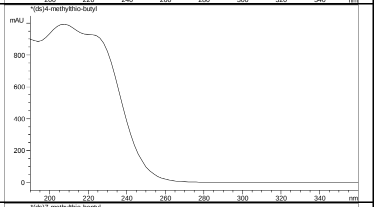
7-methylthioheptyl GS	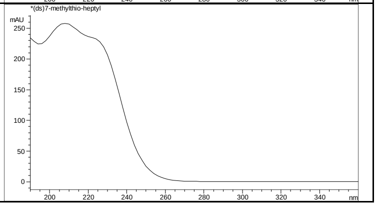

8-methylthiooctyl GS	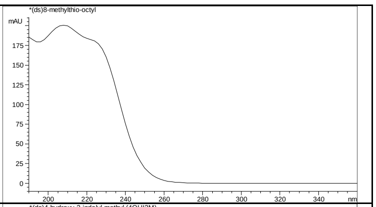
4-hydroxy-indol-3-yl-methyl GS	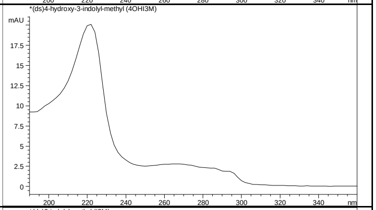
indol-3-yl-methyl GS	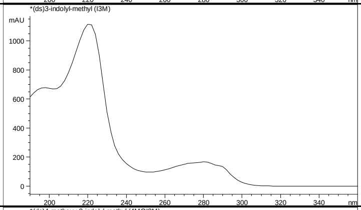
4-methoxy-indol-3-yl-methyl GS	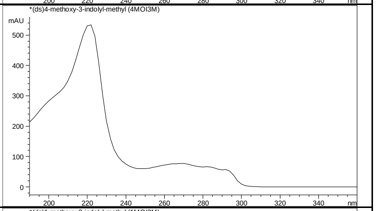
1-methoxy-indol-3-yl-methyl GS	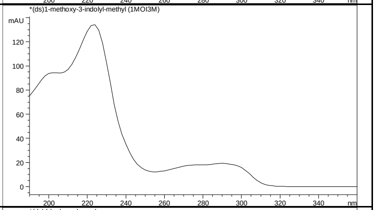
4-hydroxybenzyl GS<br>(internal standard)	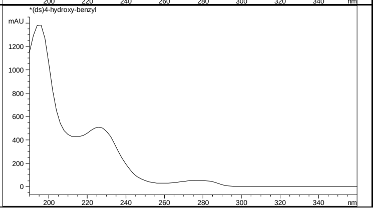
