# Supplementary material for: Water Stress and Aphid Feeding Differentially Influence Metabolite Composition in Arabidopsis thaliana (L.)
Source: PLoS One. 2012 Nov 7;7(11):e48661. doi: 10.1371/journal.pone.0048661 (PMC3492492; doi:10.1371/journal.pone.0048661)
Supplement: Table S2 — Primers list of genes associated with glucosinolate, camalexin biosynthesis or signaling pathways. (PDF) [file pone.0048661.s002.pdf]

Table S2. Primers list of genes associated with glucosinolate, camalexin biosynthesis or signalling pathways.

Gene	AGI no.	Primers
BCAT4	At3g19710	(f) 5´-TGTACTGGCACTGCTTCCA<br>(r) 5´-TCAATCTCCACCGTCCATC
MAM1	At5g23010	(f) 5´-AGAATCACGGATGCTGATTTG<br>(r) 5´-GGAAATTGATATTACTGTGGTACAC
MAM3	At5g23020	(f) 5´-AATCACAGACGCTGATCTGAA<br>(r) 5´-TGACTTAATACCATCGTAGTACCA
CYP79F1	At1g16410	(f) 5´-AGTTCCGTCCTTAAGAAGAAGA<br>(r) 5´-TTATTATGAAGTGACGGAGATTC
CYP79F2	At1g16400	(f) 5´-GCTAAGCCTCTTCTTTTGTCTGT<br>(r) 5´-TCATGATTAAGTTACGGAGATACA
CYP83A1	At4g13770	(f) 5´-GCGAACCTTCTCCTCAGC<br>(r) 5´-GAGAGACGATTGCCATTTAAG
UGT74C1	At2g31790	(f) 5´-AGGAGGTAGTTCCGACAAGA<br>(r) 5´-TCATATATGGATTTGTTGGGAG
CYP79B2	At4g39950	(f) 5´-CACTTGGAAGCTACCTGAGA<br>(r) 5´-GAACCAAGAAACCAACACACA
CYP79B3	At2g22330	(f) 5´-TTAAGTGGAAACTAGCAGGAAG<br>(r) 5´-CAAAAGGACCAAAACCGAAC
CYP83B1	At4g31500	(f) 5´-TGGGATTGCAATGGTAGAGA<br>(r) 5´-CATTGGTCACGCCATATCTAC
UGT74B1	At1g24100	(f) 5´-GAAAGGAGTGATGGAAGGAG<br>(r) 5´-TTGAATCACAGTCATCGTGGT
SUR1 (C-S-Lyase)	At2g20610	(f) 5´-GTTGGAGCTGTCATTGATGGA<br>(r) 5´-GCACACACATCCTCTTCTGT
TGG1	AT5G26000	(f) 5´-GCTCCTTATCTATGGAGCAAG<br>(r) 5´-ACGTTACCGACGAAGACTCT
CaEF	At2g33380	(f) 5´-GTAACCGAGGGAAATCGAATG<br>(r) 5´-TCCCCAAACTGAATAACAAGAC
BGL1	At1g52400	(f) 5´-CGAGGTTCGGACTTTACTACA<br>(r) 5´-CGAACAACTCACAAGTCCTTC
PR1	At2g14610	(f) 5´-AGTCAGTGAGACTCGGATGTG<br>(r) 5´-CATCCTGCATATGATGCTCCT
PAD3 (CYP71B15)	AT3G26830	(f) 5´-AGGAGGAAGCCAAAGAGATC<br>(r) 5´-AGCTGACTCCAACTGGATCA
MYB28	At5g61420	(f) 5´-GACTTCTTGGGAAACATCGG<br>(r) 5´-CACTGAGCAGATTCGCAATG
MYB29	At5g07690	(f) 5´-AATACTGGAGGAGGATATAACC<br>(r) 5´-GTTCTTGTCGTCATAATCTTGG
MYB51	At1g18570	(f) 5´-AAGTGTTTCCGTTGACTCTGAA<br>(r) 5´-AAATTATCGCAGTACATTAGAGGA
MYB34	AT5G60890	(f) 5´-CTTCGGCGACGGTGGAGTTTCC<br>(r) 5´-ATGCAATCACTCACAAACCGA
MYB76	AT5G07700	(f) 5´-TCGGTACGAGCATTGATCTC<br>(r) 5´-TGGGATGGTCAAGAAGATAAG
MYB122	AT1G74080	(f) 5´-CGAGACGACGTCGTTTAATG<br>(r) 5´-CGGTCGAAACACACTACACA
